# 2-{[2-(4-Chloro­phen­yl)hydrazinyl­idene]meth­yl}phenol

**DOI:** 10.1107/S1600536811017958

**Published:** 2011-05-20

**Authors:** Kong Mun Lo, Seik Weng Ng

**Affiliations:** a Department of Chemistry, University of Malaya, 50603 Kuala Lumpur, Malaysia

## Abstract

In the title Schiff base mol­ecule, C_13_H_11_ClN_2_O, the non-H atoms are approximately coplanar (r.m.s. deviation = 0.115 Å) and the two benzene rings are twisted by 9.36 (3)° with respect to each other. The hy­droxy group is hydrogen bonded to the azomethine N atom. In the crystal, an N—H⋯π inter­action is observed between the imino group and the hy­droxy­benzene ring of an adjacent mol­ecule.

## Related literature

For the synthesis of the compound, see: Auwers (1909 ▶).
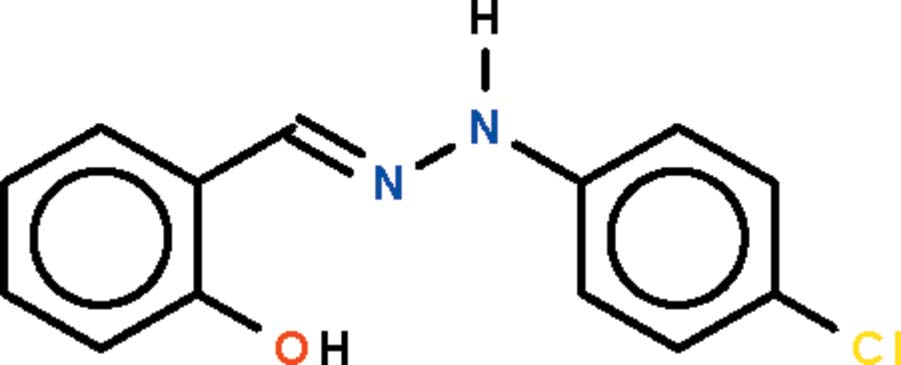


## Experimental

### 

#### Crystal data

C_13_H_11_ClN_2_O
*M* *_r_* = 246.69
Orthorhombic, 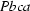

*a* = 10.7590 (1) Å
*b* = 7.3189 (1) Å
*c* = 28.9222 (3) Å
*V* = 2277.45 (4) Å^3^
*Z* = 8
Mo *K*α radiation
μ = 0.32 mm^−1^
*T* = 100 K
0.25 × 0.25 × 0.15 mm

#### Data collection

Bruker SMART APEX diffractometer
Absorption correction: multi-scan (*SADABS*; Sheldrick, 1996 ▶) *T*_min_ = 0.925, *T*_max_ = 0.954
20107 measured reflections
2614 independent reflections
2326 reflections with *I* > 2σ(*I*)
*R*_int_ = 0.033

#### Refinement

*R*[*F* ^2^ > 2σ(*F* ^2^)] = 0.031
*wR*(*F* ^2^) = 0.089
*S* = 1.04
2614 reflections
162 parameters
H atoms treated by a mixture of independent and constrained refinement
Δρ_max_ = 0.37 e Å^−3^
Δρ_min_ = −0.19 e Å^−3^

### 

Data collection: *APEX2* (Bruker, 2009 ▶); cell refinement: *SAINT* (Bruker, 2009 ▶); data reduction: *SAINT*; program(s) used to solve structure: *SHELXS97* (Sheldrick, 2008 ▶); program(s) used to refine structure: *SHELXL97* (Sheldrick, 2008 ▶); molecular graphics: *X-SEED* (Barbour, 2001 ▶); software used to prepare material for publication: *publCIF* (Westrip, 2010 ▶).

## Supplementary Material

Crystal structure: contains datablocks global, I. DOI: 10.1107/S1600536811017958/xu5209sup1.cif

Structure factors: contains datablocks I. DOI: 10.1107/S1600536811017958/xu5209Isup2.hkl

Supplementary material file. DOI: 10.1107/S1600536811017958/xu5209Isup3.cml

Additional supplementary materials:  crystallographic information; 3D view; checkCIF report

## Figures and Tables

**Table 1:** Hydrogen-bond geometry (Å, °) *Cg*1 is the centroid of the C1–C6 benzene ring.

*D*—H⋯*A*	*D*—H	H⋯*A*	*D*⋯*A*	*D*—H⋯*A*
O1—H1⋯N1	0.84 (2)	1.88 (2)	2.6382 (13)	149.8 (19)
N2—H2⋯*Cg*1^i^	0.859 (18)	2.73 (2)	3.3675 (12)	132.3 (17)

Symmetry code: (i) 
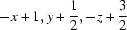
.

**Fig. 1.**
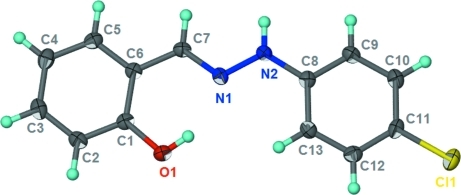
Thermal ellipsoid plot (Barbour, 2001) of C13H11ClN2O at the 70% probability level; hydrogen atoms are drawn as spheres of arbitrary radius.

## supplementary crystallographic information

### Comment

There in a enormous amount of literature on Schiff bases, which are synthesized by reaction of a primary amine with a carbonyl function. The synthesis of the title hydrazone (Scheme I) was reported a century ago (Auwers, 1909). The C_13_H_11_ClN_2_O molecule (Scheme I) is twisted along the –CH═N–NH– portion that connects the two aromatic rings. The non-hydrogen atoms approximately lie on plane (r.m.s. deviation 0.115 Å), the rings being twisted by 9.36 (3)°. The hydroxy group is hydrogen-bond donor the the azomethine N atom (Fig. 1). The amino H atom is involved in the N—H···π interaction in the crystal structure (Table 1).

### Experimental

Salicylaldehyde (1 ml, 10 mmol) and 4-chlorophenylhydrazine hydrochloride (1.8 g, 10 mmol) were dissolved in ethanol (100 ml). No HCl-abstracting reagent was added. The solution was heated for an hour. Slow evaporation of the solvent gave colorless crystals of the Schiff base.

### Refinement

Carbon-bound H atoms were placed in calculated positions (C—H 0.95 Å) and were included in the refinement in the riding model approximation, with *U*(H) set to 1.2 times *U*_eq_(C). The amino and hydroxy H atoms were located in a difference Fourier map, and were freely refined.

### Crystal data

**Table:**

C_13_H_11_ClN_2_O	*F*(000) = 1024
*M**_r_* = 246.69	*D*_x_ = 1.439 Mg m^−^^3^
Orthorhombic, *P**b**c**a*	Mo *K*α radiation, λ = 0.71073 Å
Hall symbol: -P 2ac 2ab	Cell parameters from 7363 reflections
*a* = 10.7590 (1) Å	θ = 2.4–28.3°
*b* = 7.3189 (1) Å	µ = 0.32 mm^−^^1^
*c* = 28.9222 (3) Å	*T* = 100 K
*V* = 2277.45 (4) Å^3^	Block, colorless
*Z* = 8	0.25 × 0.25 × 0.15 mm

### Data collection

**Table:**

Bruker SMART APEX diffractometer	2614 independent reflections
Radiation source: fine-focus sealed tube	2326 reflections with *I* > 2σ(*I*)
graphite	*R*_int_ = 0.033
ω scans	θ_max_ = 27.5°, θ_min_ = 2.4°
Absorption correction: multi-scan (*SADABS*; Sheldrick, 1996)	*h* = −13→13
*T*_min_ = 0.925, *T*_max_ = 0.954	*k* = −9→9
20107 measured reflections	*l* = −37→37

### Refinement

**Table:**

Refinement on *F*^2^	Primary atom site location: structure-invariant direct methods
Least-squares matrix: full	Secondary atom site location: difference Fourier map
*R*[*F*^2^ > 2σ(*F*^2^)] = 0.031	Hydrogen site location: inferred from neighbouring sites
*wR*(*F*^2^) = 0.089	H atoms treated by a mixture of independent and constrained refinement
*S* = 1.04	*w* = 1/[σ^2^(*F*_o_^2^) + (0.0487*P*)^2^ + 0.9769*P*] where *P* = (*F*_o_^2^ + 2*F*_c_^2^)/3
2614 reflections	(Δ/σ)_max_ = 0.001
162 parameters	Δρ_max_ = 0.37 e Å^−^^3^
0 restraints	Δρ_min_ = −0.19 e Å^−^^3^

### Special details

**Table:**

Geometry. All e.s.d.'s (except the e.s.d. in the dihedral angle between two l.s. planes) are estimated using the full covariance matrix. The cell e.s.d.'s are taken into account individually in the estimation of e.s.d.'s in distances, angles and torsion angles; correlations between e.s.d.'s in cell parameters are only used when they are defined by crystal symmetry. An approximate (isotropic) treatment of cell e.s.d.'s is used for estimating e.s.d.'s involving l.s. planes.

### Fractional atomic coordinates and isotropic or equivalent isotropic displacement parameters (Å^2^)

**Table:**

	*x*	*y*	*z*	*U*_iso_*/*U*_eq_	
Cl1	0.72028 (3)	0.56809 (4)	0.484943 (10)	0.02413 (12)	
O1	0.75260 (9)	0.62244 (14)	0.75810 (3)	0.0202 (2)	
N1	0.57723 (9)	0.76810 (14)	0.70608 (3)	0.0160 (2)	
N2	0.52407 (10)	0.81449 (15)	0.66514 (4)	0.0192 (2)	
C1	0.68256 (11)	0.67666 (16)	0.79476 (4)	0.0158 (2)	
C2	0.72583 (11)	0.63711 (18)	0.83900 (4)	0.0183 (3)	
H2A	0.8022	0.5739	0.8429	0.022*	
C3	0.65750 (12)	0.68996 (16)	0.87755 (4)	0.0197 (3)	
H3	0.6883	0.6646	0.9077	0.024*	
C4	0.54397 (12)	0.77994 (17)	0.87237 (4)	0.0198 (3)	
H4	0.4971	0.8152	0.8987	0.024*	
C5	0.50049 (12)	0.81715 (16)	0.82826 (4)	0.0174 (2)	
H5	0.4230	0.8778	0.8247	0.021*	
C6	0.56790 (11)	0.76755 (16)	0.78874 (4)	0.0150 (2)	
C7	0.51856 (11)	0.81180 (16)	0.74331 (4)	0.0157 (2)	
H7	0.4414	0.8743	0.7410	0.019*	
C8	0.57272 (11)	0.75285 (16)	0.62353 (4)	0.0157 (2)	
C9	0.50203 (11)	0.77689 (17)	0.58344 (4)	0.0183 (3)	
H9	0.4223	0.8322	0.5854	0.022*	
C10	0.54686 (12)	0.72102 (17)	0.54082 (4)	0.0193 (3)	
H10	0.4986	0.7382	0.5137	0.023*	
C11	0.66308 (12)	0.63963 (17)	0.53830 (4)	0.0180 (2)	
C12	0.73438 (12)	0.61342 (17)	0.57764 (4)	0.0182 (3)	
H12	0.8137	0.5569	0.5754	0.022*	
C13	0.68960 (11)	0.66998 (16)	0.62040 (4)	0.0171 (2)	
H13	0.7383	0.6524	0.6474	0.020*	
H1	0.7170 (18)	0.662 (3)	0.7343 (7)	0.047 (6)*	
H2	0.4532 (17)	0.868 (3)	0.6662 (6)	0.032 (5)*	

### Atomic displacement parameters (Å^2^)

**Table:**

	*U*^11^	*U*^22^	*U*^33^	*U*^12^	*U*^13^	*U*^23^
Cl1	0.0330 (2)	0.02229 (18)	0.01713 (17)	0.00084 (13)	0.00504 (12)	−0.00208 (11)
O1	0.0173 (4)	0.0242 (5)	0.0191 (5)	0.0049 (4)	0.0013 (4)	−0.0008 (4)
N1	0.0172 (5)	0.0153 (5)	0.0154 (5)	−0.0010 (4)	−0.0023 (4)	0.0006 (4)
N2	0.0184 (5)	0.0240 (6)	0.0152 (5)	0.0063 (4)	−0.0014 (4)	0.0001 (4)
C1	0.0158 (6)	0.0126 (5)	0.0189 (6)	−0.0020 (4)	0.0007 (4)	−0.0014 (4)
C2	0.0186 (6)	0.0145 (6)	0.0218 (6)	−0.0009 (5)	−0.0036 (5)	0.0005 (5)
C3	0.0260 (6)	0.0158 (6)	0.0171 (6)	−0.0041 (5)	−0.0036 (5)	0.0014 (4)
C4	0.0250 (6)	0.0177 (6)	0.0166 (6)	−0.0040 (5)	0.0034 (5)	−0.0009 (5)
C5	0.0172 (6)	0.0143 (5)	0.0208 (6)	−0.0004 (5)	0.0026 (5)	−0.0005 (4)
C6	0.0155 (5)	0.0121 (5)	0.0174 (6)	−0.0022 (4)	−0.0004 (4)	0.0000 (4)
C7	0.0141 (6)	0.0133 (5)	0.0195 (6)	−0.0001 (4)	0.0002 (4)	0.0005 (4)
C8	0.0170 (6)	0.0139 (5)	0.0162 (6)	−0.0025 (4)	0.0007 (4)	0.0012 (4)
C9	0.0166 (6)	0.0185 (6)	0.0199 (6)	0.0001 (5)	−0.0021 (5)	0.0012 (5)
C10	0.0217 (6)	0.0194 (6)	0.0169 (6)	−0.0022 (5)	−0.0033 (5)	0.0010 (5)
C11	0.0241 (6)	0.0152 (5)	0.0147 (5)	−0.0024 (5)	0.0038 (5)	−0.0010 (4)
C12	0.0185 (6)	0.0153 (6)	0.0210 (6)	0.0008 (5)	0.0024 (5)	0.0024 (5)
C13	0.0175 (6)	0.0167 (6)	0.0171 (6)	−0.0006 (5)	−0.0016 (4)	0.0022 (4)

### Geometric parameters (Å, °)

**Table:**

Cl1—C11	1.7418 (12)	C5—C6	1.4014 (16)
O1—C1	1.3600 (15)	C5—H5	0.9500
O1—H1	0.84 (2)	C6—C7	1.4539 (16)
N1—C7	1.2883 (15)	C7—H7	0.9500
N1—N2	1.3580 (14)	C8—C9	1.3978 (16)
N2—C8	1.3878 (15)	C8—C13	1.3991 (17)
N2—H2	0.859 (18)	C9—C10	1.3855 (17)
C1—C2	1.3919 (17)	C9—H9	0.9500
C1—C6	1.4123 (17)	C10—C11	1.3870 (18)
C2—C3	1.3905 (18)	C10—H10	0.9500
C2—H2A	0.9500	C11—C12	1.3857 (18)
C3—C4	1.3958 (19)	C12—C13	1.3901 (17)
C3—H3	0.9500	C12—H12	0.9500
C4—C5	1.3859 (17)	C13—H13	0.9500
C4—H4	0.9500		
			
C1—O1—H1	106.7 (14)	C1—C6—C7	122.36 (11)
C7—N1—N2	117.40 (10)	N1—C7—C6	121.41 (11)
N1—N2—C8	121.06 (10)	N1—C7—H7	119.3
N1—N2—H2	117.2 (11)	C6—C7—H7	119.3
C8—N2—H2	121.0 (11)	N2—C8—C9	118.24 (11)
O1—C1—C2	118.08 (11)	N2—C8—C13	122.42 (11)
O1—C1—C6	121.69 (11)	C9—C8—C13	119.33 (11)
C2—C1—C6	120.22 (11)	C10—C9—C8	120.76 (11)
C1—C2—C3	120.17 (11)	C10—C9—H9	119.6
C1—C2—H2A	119.9	C8—C9—H9	119.6
C3—C2—H2A	119.9	C9—C10—C11	119.17 (11)
C2—C3—C4	120.51 (11)	C9—C10—H10	120.4
C2—C3—H3	119.7	C11—C10—H10	120.4
C4—C3—H3	119.7	C10—C11—C12	121.02 (11)
C5—C4—C3	119.15 (11)	C10—C11—Cl1	119.62 (9)
C5—C4—H4	120.4	C12—C11—Cl1	119.36 (10)
C3—C4—H4	120.4	C11—C12—C13	119.83 (12)
C4—C5—C6	121.68 (11)	C11—C12—H12	120.1
C4—C5—H5	119.2	C13—C12—H12	120.1
C6—C5—H5	119.2	C12—C13—C8	119.88 (11)
C5—C6—C1	118.27 (11)	C12—C13—H13	120.1
C5—C6—C7	119.37 (11)	C8—C13—H13	120.1
			
C7—N1—N2—C8	−171.80 (11)	C1—C6—C7—N1	0.68 (18)
O1—C1—C2—C3	179.94 (11)	N1—N2—C8—C9	168.57 (11)
C6—C1—C2—C3	−1.21 (19)	N1—N2—C8—C13	−12.10 (18)
C1—C2—C3—C4	1.21 (19)	N2—C8—C9—C10	178.83 (12)
C2—C3—C4—C5	−0.44 (18)	C13—C8—C9—C10	−0.52 (18)
C3—C4—C5—C6	−0.32 (18)	C8—C9—C10—C11	0.32 (19)
C4—C5—C6—C1	0.31 (18)	C9—C10—C11—C12	0.10 (19)
C4—C5—C6—C7	−179.31 (11)	C9—C10—C11—Cl1	179.78 (10)
O1—C1—C6—C5	179.26 (11)	C10—C11—C12—C13	−0.30 (19)
C2—C1—C6—C5	0.46 (18)	Cl1—C11—C12—C13	−179.99 (10)
O1—C1—C6—C7	−1.13 (18)	C11—C12—C13—C8	0.10 (18)
C2—C1—C6—C7	−179.93 (11)	N2—C8—C13—C12	−179.01 (11)
N2—N1—C7—C6	179.85 (10)	C9—C8—C13—C12	0.31 (18)
C5—C6—C7—N1	−179.72 (11)		

### Hydrogen-bond geometry (Å, °)

**Table:**

Cg1 is the centroid of the C1–C6 benzene ring.

**Table:**

*D*—H···*A*	*D*—H	H···*A*	*D*···*A*	*D*—H···*A*
O1—H1···N1	0.84 (2)	1.88 (2)	2.6382 (13)	149.8 (19)
N2—H2···Cg1^i^	0.859 (18)	2.73 (2)	3.3675 (12)	132.3 (17)

Symmetry codes: (i) −*x*+1, *y*+1/2, −*z*+3/2.
